# A Good Short-term Outcome in Delayed Decompression of Cauda Equina Syndrome in *Klebsiella pneumoniae* Spinal Epidural Abscess: A Case Report

**DOI:** 10.5704/MOJ.1707.018

**Published:** 2017-07

**Authors:** J Hanifah, J Joehaimey, MI Yusof

**Affiliations:** Department of Orthopaedics, Universiti Sains Malaysia, Kubang Kerian, Malaysia

**Keywords:** spinal epidural abscess, klebsiella pneumoniae, cauda equina syndrome, delayed decompression

## Abstract

Spinal epidural abscess is a severe, generally pyogenic, infection of the epidural space of spinal cord or cauda equina. The swelling caused by the abscess leads to compression or vascular disruption of neurological structures that requires urgent surgical decompression to avoid significant permanent disability. We share a rare case of *Klebsiella pneumoniae* spinal epidural abscess secondary to haematogenous spread of previous lung infection that presented late at our centre with cauda equina syndrome that showed good short-term outcome in delayed decompression. A 50-year old female presented with one-week history of persistent low back pain with progressively worsening bilateral lower limb weakness for seven days and urinary retention associated with saddle anesthesia of 2-day duration. Magnetic resonance imaging with contrast of the lumbo-sacral region showed an intramuscular collection of abscess at left gluteus maximus and left multifidus muscle with a L3-L5 posteriorly placed extradural lesion enhancing peripherally on contrast, suggestive of epidural abscess that compressed the cauda equina. The pus was drained using the posterior lumbar approach. Tissue and pus culture revealed *Klebsiella pneumoniae*, suggestive of bacterial infection. The patient made immediate improvement of muscle power over bilateral lower limbs postoperative followed by ability to control micturition and defecation the 4^th^ post-operative day. A good short-term outcome in delayed decompression of cauda equine syndrome is extremely rare. Aggressive surgical decompression combined with antibiotic therapy led to good short-term outcome in this patient despite delayed decompression of more than 48 hours.

## Introduction

*Klebsiella pneumoniae* is a rare cause of infection in spinal epidural abscess that may rarely develop by haematogenous spread from any primary focus. Early diagnosis, prompt decompression and pus drainage in cauda equina syndrome within 48 hours, and antibiotic therapy remain the important predictors of successful neurological outcome for a pyogenic spinal epidural abscess complicated by cauda syndrome. Many literatures show poor outcome if surgical decompression of cauda syndrome done after a period of 48 hours. Here, we share a rare case of a good short-term outcome in delayed decompression of cauda equina syndrome secondary to *Klebsiella pneumoniae* spinal epidural abscess.

## Case Report

We report the case of a 50-year old female patient with underlying poorly controlled diabetes mellitus who presented with a history of persistent low back pain with progressively worsening bilateral lower limb weakness for seven days causing her inability to ambulate and with urinary retention associated with saddle anesthesia of 2-day duration. These complaints were associated with history of loss of appetite and weight for the past three months prior to admission. Three months previously, she gave a history of lung infection for which she was admitted to the hospital for intravenous antibiotics for one week. There was no contact history with tuberculosis patient. Skin test for tuberculosis and sputum for tuberculosis organism were negative.

At initial presentation at our emergency department, the patient was looking lethargic. She had no fever, and no history of trauma. Physical examination revealed normal blood pressure, pulse and respiratory rate. Chest and abdominal examinations showed no abnormality. There was tenderness over the left loin and gluteal region. Otherwise, no deformity, swelling or discharging sinus were noted. Neurological examination revealed hypotonia at both lower limbs, with power of grade 3/5. Deep tendon reflexes were reduced in both lower limbs. The anal tone was lax with reduced perianal sensation and absent bulbocavernosus reflex.

**Fig. 1:**
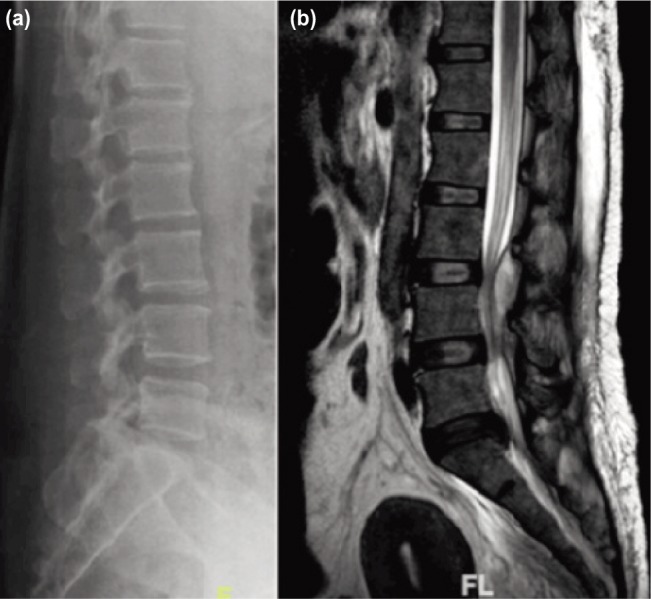
(a) Preoperative plain radiograph lumbo-sacral region, showing no bony involvement. Vertebral bony height and disc spaces were normal, and (b) T2-weighted magnetic resonance imaging sagittal view, showing elongated, diffuse, multisegmented abscess collection causing compression of intraspinal nerve roots at L3/L4 and L4/L5 (cauda equina).

**Fig. 2:**
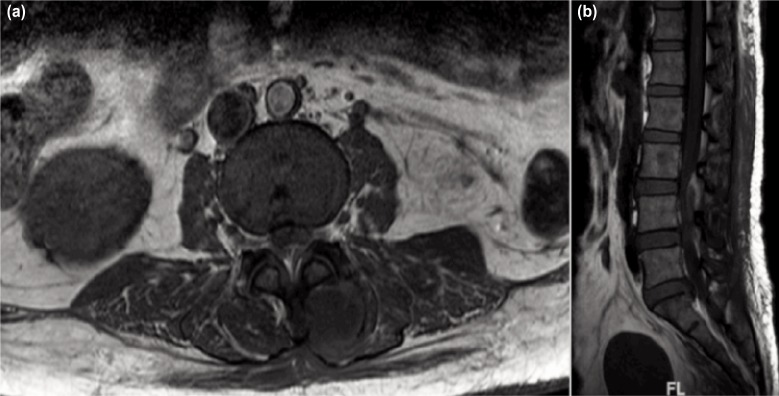
(a) Axial view MRI; The lesion was hypointense on T1-weighted magnetic resonance imaging and was enhancing peripherally on contrast, suggestive of epidural abscess and (b) Sagittal view MRI; The lesion was hypointense on T1-weighted magnetic resonance imaging and was enhancing peripherally on contrast, suggestive of epidural abscess.

Initial laboratory investigations showed raised total leukocyte count of 16.3 × 10^9^/L, raised erythrocyte sedimentation rate of 102 mm/h and raised C-reactive protein of 92 mg/dL. The plain radiograph of the lumbosacral region did not show any bony involvement and intervertebral disc involvement (Fig. 1a). Chest radiograph was normal. Magnetic resonance imaging with contrast of the lumbo-sacral region showed intramuscular abscess collection at left gluteus maximus and left multifidus muscle with a L3-L5 posteriorly placed extradural lesion causing compression of intraspinal nerve root at L3/L4 and L4/L5 (cauda equina) (Fig. 1b). The lesion was hypointense on TI-weighted image, hyperintense on T2-weighted image, not suppressed in fat sequence and was enhancing peripherally on contrast, suggestive of epidural abscess (Fig. 1 and 2).

**Fig. 3:**
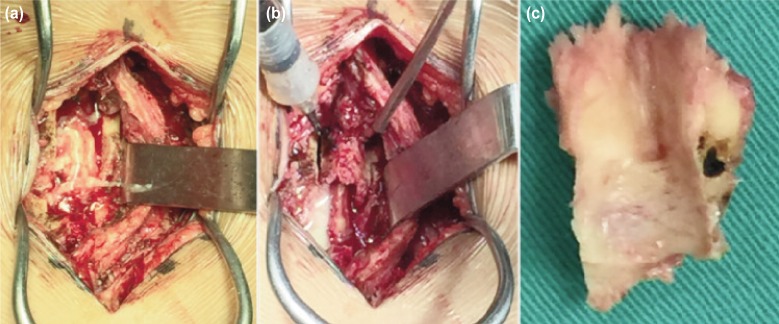
(a) Pus collection noted over paravertebral muscles extending from L3 to L5 lamina, (b) Surgical posterior decompression by laminectomy both lamina at the level of L3 until L5 and drainage of abscess and (c) Both lamina and interspinous process at the level of L3 until L5 post posterior decompression.

The patient underwent urgent surgical posterior decompression and drainage of abscess. (Fig. 3). Intraoperative, there were two circumscribed loculi of deep epidural abscess over the paravertebral muscle extending from L3 to L5 lamina with intramuscular abscess over the left gluteal region. Surprisingly, postoperative, she showed immediate improvement of muscle power over both lower limbs, followed by ability to control micturition and defecation after 4th post-operation day. Culture of the pus yielded *Klebsiella pneumoniae*. The antibiotic was then, changed per culture specific antibiotic agents. At time of discharge, the power was 4/5 over L2-L3 myotome and 5/5 over L4-S1 myotome in both lower limbs with ability to ambulate again using walking frame, and she was continent to bladder and bowel.

## Discussion

Spinal epidural abscess (SEA) is primarily a bacterial infection commonly caused by *Staphylococcus aureus* organism^1^. It is typically associated with a disc space infection or vertebral osteomyelitis because of contiguous spread of pathogens from adjacent bone or disc which is the common route of infection. However, haematogenous spread into the isolated epidural space without bony involvement may also occur from another distant source of infection. Risk factors for infection include diabetes mellitus, previous back trauma, intravenous drug use and pregnancy. Studies have also documented that spinal epidural abscess occurs because of direct inoculation at the time of operation, epidural steroid injection, lumbar puncture, and epidural catheterization^2^.

In the present case, the patient had a history of severe lung infection three months prior to admission. We postulate that haematogenous spread had occurred to spinal epidural space during the previous lung infection. In addition, because the patient was immunocompromised due to poorly controlled diabetes mellitus, the probability of the haematogenous spread occurring was high. The patient also gave history of loss of appetite and weight for the past three months despite apparent full recovery from the lung infection after one week course of intravenous antibiotics.

Surgical intervention remains the mainstay of treatment for the pyogenic epidural abscess. Prompt decompression of the spinal cord and neural structures, followed by culture-specific antimicrobial treatments is the recommended treatment in patients with significant neurological deficit and solitary epidural abscess without bony involvement. However, the most significant prognostic factor in patients with pyogenic epidural abscess is the duration of paraplegia. Patel *et al* retrospectively reviewed 128 cases of spinal epidural abscess and concluded that four major risk factors associated with failure of medical management are diabetes mellitus, CRP>115 mg/L, WBC>12.5 × 10^9^ cells/L, and positive blood cultures (bacteraemia)^3^. However, almost all authors concluded that there was no role for non-operative treatment in patients presenting with worsening neurologic symptoms or progressive disease.

Cauda equina syndrome (CES) is a rare orthopaedic emergency disorder that needs urgent surgical decompression. Delay in surgical intervention can lead to permanent paralysis, impaired bladder or bowel control and other associated problems. Cauda equina syndrome caused by spinal epidural infection, as it presented in our case, is rare and can have poor prognosis even after surgical decompression because of the increased probability of developing postoperative complications, such as arachnoiditis^4^. Many authors recommend emergency surgical debridement if onset of complete spinal cord injury is less than 48 hours. Emergency surgery performed within 48 hours on patients who present with acute complete loss of neurological function secondary to SEA have been reported in multiple reports to have a good recovery^4,5^. However, the exact time point when a neurological injury becomes irreversible is unknown.

We report a rare case of spinal abscess with cauda equina syndrome complicating *Klebsiella pneumoniea* lung infection that showed good short-term outcome even after delayed decompression. Although, timing of the decompression was suggestive to be as soon as possible preferably within 48 hours, in our opinion delayed aggressive decompression more than 48 hours can still give good outcome with ability to regain walking ability, urinary and bowel function but should be reserved only for selected patients presenting late.

## References

[1] Chen HC, Tzaan WC, Lui TN (2004). Spinal epidural abscesses: a retrospective analysis of clinical manifestations, sources of infection, and outcomes. Chang Gung Med J.

[2] Danner RL, Hartman BJ (1987). Update on spinal epidural abscess: 35 cases and review of the literature. Rev Infect Dis.

[3] Tuchman A, Pham M, Hsieh PC (2014). The indications and timing for operative management of spinal epidural abscess: literature review and treatment algorithm. Neurosurg Focus.

[4] Patel AR, Alton TB, Bransford RJ, Lee MJ, Bellabarba CB, Chapman JR (2014). Spinal epidural abscesses: Risk factors, medical versus surgical management, a retrospective review of 128 cases. Spine J.

[5] Turgut M (2008). Complete recovery of acute paraplegia due to pyogenic thoracic spondylodiscitis with an epidural abscess. Acta Neurochir (Wien).

